# Aging and Complexity Effects on Hemisphere-Dependent Movement-Related Beta Desynchronization during Bimanual Motor Planning and Execution

**DOI:** 10.3390/brainsci12111444

**Published:** 2022-10-26

**Authors:** Sybren Van Hoornweder, Diego Andres Blanco-Mora, Siel Depestele, Kim van Dun, Koen Cuypers, Stefanie Verstraelen, Raf Meesen

**Affiliations:** 1 REVAL—Rehabilitation Research Center, Faculty of Rehabilitation Sciences, University of Hasselt, 3590 Diepenbeek, Belgium; 2 Faculty of Medicine, University of Lisbon, 1000 Lisbon, Portugal; 3 Movement Control and Neuroplasticity Research Group, Group Biomedical Sciences, Department of Movement Sciences, KU Leuven, 3000 Leuven, Belgium; 4 Leuven Brain Institute (KU Leuven-LBI), 3001 Leuven, Belgium

**Keywords:** electroencephalography, aging, bimanual coordination, interlimb coordination, motor planning, motor execution, beta oscillations, time-frequency analysis

## Abstract

With aging comes degradation of bimanual movement performance. A hallmark feature of bimanual movements is movement-related beta desynchronization (MRBD), an attenuation in the amplitude of beta oscillations associated with sensorimotor activation. Here, we investigated MRBD in 39 healthy adults (20 younger and 19 older adults) in frontal, central, and parietal regions across both hemispheres, during the planning and execution of a bimanual tracking task. Task accuracy decreased with age and during more difficult conditions when both hands had to move at different relative speeds. MRBD was mostly situated in the central region, and increased in older versus younger adults during movement execution but not planning. Irrespective of age, motor planning and execution were associated with increased MRBD in the left and right hemispheres, respectively. Notably, right central MRBD during motor planning was associated with bimanual task performance, particularly in older adults. Specifically, persons who demonstrated high MRBD during motor planning performed better on the bimanual tracking task. Our results highlight the importance of lateralized MRBD during motor planning, thereby shining new light on previous research and providing a promising avenue for future interventions.

## 1. Introduction

Bimanual coordination encompasses a diverse group of motor behaviors wherein the brain simultaneously coordinates both hands in order to adequately perform an activity. Despite its ubiquity, bimanual coordination deteriorates with age [1]. As bimanual movements take place approximately two times more than unimanual movements in daily life, this deterioration introduces detrimental functional impairments into the lives of older adults [2].

Magneto- and electroencephalography (EEG) studies consistently demonstrate that modulations in neural oscillatory activity in the beta frequency band (13–30 Hz) are a hallmark feature of bimanual movements, and motor behavior in general [3,4,5,6,7]. Specifically, attenuation of sensorimotor beta power during motor planning and execution, also known as movement-related beta desynchronization (MRBD), is a distinct trait of motor behavior [8]. Several functional magnetic resonance imaging and transcranial magnetic stimulation (TMS) studies suggest that MRBD is associated with elevated blood-oxygenated level dependent (BOLD) signal and cortical excitability, respectively, providing support for the hypothesis that MRBD resembles sensorimotor network activation [9,10,11,12,13,14]. Despite its clear affiliation to the sensorimotor network, the current fundamental and functional understanding of MRBD remains fragmentary, with the majority of studies focusing on unimanual movements [8,15,16,17].

Analogous to bimanual control, age and motor complexity affect MRBD [5,6,7,18,19,20,21].

Regarding age, MRBD is known to increase across the lifespan [6,7,18,19,20]. Likely, this serves to compensate for the age-related elevation of resting state beta power, to ensure that the absolute beta power level of older adults during movement execution is similar to that of younger adults [6,7]. For instance, Protzak and Gramann (2021) observed more pronounced MRBD in older versus younger adults during a button-press task performed while sitting and walking. Likewise, we previously observed an age-related MRBD increase during a complex four limb reaction time task [18,20].

Concerning motor complexity, we showed that MRBD increases along with movement complexity, whereby MRBD magnitude was positively correlated with task performance in older adults [18]. This latter observation agrees with Meziane et al. (2015), who observed more MRBD in older adults that performed well on a reaching motor task [22]. Overall, the aforementioned studies emphasize the functional relevance of MRBD and support the compensatory role of age-related cortical activity increases during complex motor behavior.

Research investigating MRBD associated with age-related changes in bimanual coordination is sparse. Blais et al. (2014) let younger and older adults perform a finger tapping task at 1 Hz with three coordination modes: an in-phase mode (left index tapped with a 0 s delay relative to right index), an anti-phase mode (left index tapped with 0.5 s delay), and an inter-phase mode (left index tapped with 0.25 s delay) [5]. As age and complexity were positively associated with MRBD, their results corroborate several studies reporting increased motor network activations during anti-phase versus in-phase movements, particularly in older adults [23,24]. Xifra-Porxas et al. (2019) assessed MRBD during a bimanual sustained handgrip task [6]. While they also observed an age-related MRBD increase, they did not find a positive association between MRBD magnitude and task performance. On the contrary, they reported higher MRBD was related to worse performance, irrespective of age [18].

While previous literature demonstrated that MRBD plays a functional role in both simple and complex movements and is modulated by age and task complexity, several important gaps in our understanding of MRBD remain. For instance, the limited research on MRBD in bimanual movements, and interlimb movements in general, does not differentiate between motor planning and execution. Doing so could be enlightening, as MRBD during unimanual movements is only related to complexity during movement planning but not execution [15]. Additionally, the role of hemispheric laterality on MRBD during bimanual movements remains unclear. While some studies report that the non-dominant (right) hemisphere shows more activation in response to increased complexity [3,21,25,26], the opposite has also been reported [27,28,29,30]. Since the degree of beta power lateralization during unimanual movements is reduced in older adults, it would be interesting to see how this translates to bimanual movements, where lateralized brain activity has also been observed [27,31,32]. Notably, investigating hemispheric lateralization in the context of bimanual motor planning and execution might be particularly informative, as the left and right sensorimotor networks have been related to, respectively, motor planning and execution [21,24,33,34,35,36,37,38].

To overcome these knowledge gaps, we investigated MRBD characteristics in younger and older adults, during the planning and execution of continuous bimanual movements with varying complexity levels. Specifically, we included the bimanual tracking task (BTT), which has been used by numerous studies, and has provided telltale insights into the spatiotemporal constraints of bimanual coordination [21,33,39,40,41]. In addition to enhancing our mechanistic understanding of MRBD, which in itself is a valuable goal, we expect that our work will be of use to future interventions aiming to diminish the age-related deterioration of bimanual motor control [42,43]. In this regard, it is encouraging that Parkinson’s disease treatments which successfully alleviate motor symptoms, also reverse beta band activity disruptions [44,45].

We formulated five hypotheses, based on previous literature. First, we hypothesize that BTT performance will be worse in older adults, and will decrease with increasing task complexity in both groups. Second, we expect that MRBD values will be elevated in older adults. Third, we hypothesize for both age groups that MRBD will be most apparent in the left (dominant) hemisphere during motor planning, and in the right (non-dominant) hemisphere during motor execution, where it will increase along with movement complexity. Fourth, we expect that hemispheric lateralization will be reduced in older adults. Fifth, we hypothesize that (absolute) MRBD values will be positively correlated with complex bimanual coordination in older adults.

## 2. Materials and Methods

### 2.1. Participants

In total, 40 healthy adults participated. One participant was excluded due to excessive EEG noise. Hence, data were analyzed from 39 participants, who were either younger (*n* = 20, aged 22.3 ± 1.0 years (mean ± standard deviation), 10 females) or older adults (*n* = 19, aged 70.7 ± 3.0 years, 8 females). Participants had (corrected to) normal vision, were strongly right-handed according to the Edinburgh Handedness Inventory (younger adults = 92.9 ± 9.2, older adults = 97.6 ± 6.0) [46], and scored ≥ 24 on the Montreal Cognitive Assessment (younger adults = 28.9 ± 1.2, older adults = 26.5 ± 1.7), which indicated the absence of mild cognitive impairment [47,48]. Exclusion criteria were: (1) playing a musical instrument; (2) smoking [49]; (3) presence of a pathological condition affecting the central nervous system; (4) usage of psycho-active medication (e.g., sedatives, anti-depressants, etc.). Participants provided written informed consent prior to participation.

### 2.2. Bimanual Tracking Task

**Figure 1:**
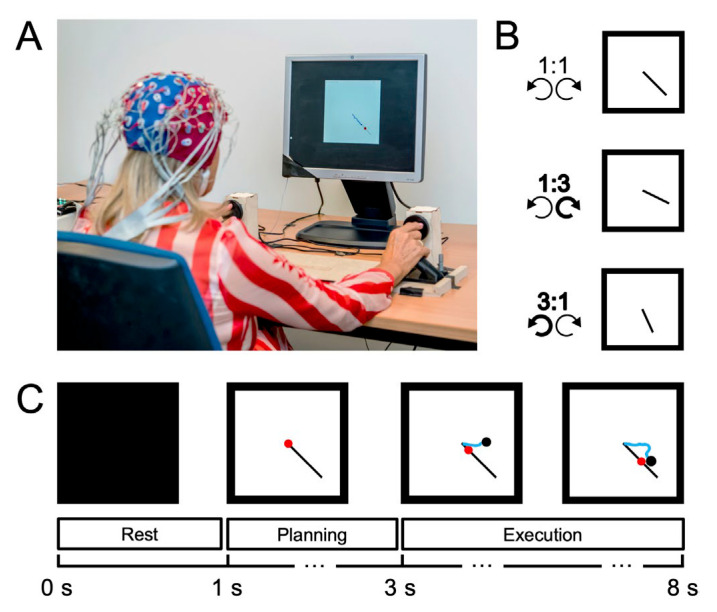
Bimanual tracking task. (**A**). Task set-up. (**B**). Task conditions, with 1:1 denoting an identical relative inter-hand frequency, and 3:1 and 1:3 denoting that the left or right hand, respectively, rotated three times faster than the other hand. (**C**). Time course of a 1:1 trial. The red and black dots denote the target and participant’s cursor location, respectively. The blue line supplies feedback about the completed trajectory.

**Figure A1:**
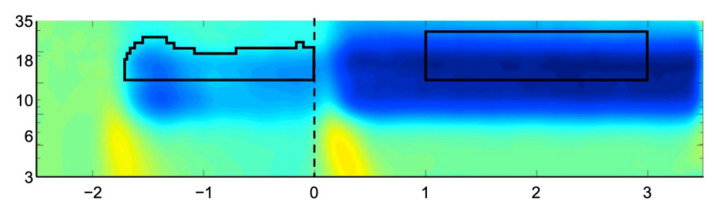
Grand-average time-frequency mask obtained by averaging the power values per time- and frequency-point, across all participants, electrodes and BTT conditions. This mask was used for power value extraction. Specifically, the mean beta power value within the black contours was extracted per participant, electrode and BTT condition and used in the statistical analyses. The black contours were defined by a statistical masking procedure (cf., Section 2.5.2. Effect of age, hemispheric laterality, and complexity on MRBD during bimanual planning and execution (Hypotheses 2–4)). Colors denote spectral power, with dark blue and red being −3 and 3 dB, respectively.

An adapted version of the BTT was used [41]. Participants were seated ~75 cm in front of a screen with their pronated forearms resting on a table (Figure 1A). A wooden frame, not shown in Figure 1A, was placed over the forearms so that participants could not see their hands. Their hands held a handlebar and each extended index finger was placed in the circular groove of a rotatable dial. The rotatable dials were connected to a shaft encoder (A1230, Allegro microsystems) for angular displacement registration (sampling frequency = 100 Hz). The BTT’s aim was to follow a moving target dot on a straight, inclined line with a cursor as accurately as possible. To do so, participants were instructed to simultaneously rotate the dials with both index fingers. Left and right dial rotations were associated with cursor movement along the ordinate and abscissa, respectively. There were three different conditions, which all required participants to rotate their hands outwards (Figure A1). The 1:1 condition required both hands to rotate at the same inter-limb frequency whereas the 1:3 and 3:1 condition required the right or left hand, respectively, to move three times faster than the other hand.

Each trial started with a rest stage (1 s), depicted by a black screen (Figure 1C). Subsequently, the planning stage (2 s) started. During this stage, a white window appeared on-screen, containing a red dot (tracking target) and a black line with a specific inclination. Depending on the BTT condition, three different lines could appear (Figure A1). All lines were situated in the lower right screen quadrant. Finally, the execution stage (5 s) started, indicated by an auditory signal (500 ms) and the start of the red dot moving over the black line at a constant speed throughout the execution stage. The participant received online feedback by means of a blue line.

BTT performance was assessed via tracking error [33,41]. A tracking error is defined as the sum of the Euclidean distance between the participant’s cursor and the red dot, and the orthogonal distance from the participant’s cursor to the target line, averaged throughout the 5 s trajectory. It is an indicator of compliance with the inquired spatiotemporal bimanual pattern, with a lower tracking error representing better performance.

Prior to BTT performance, participants received standardized task instructions. They were informed about the aim and trial structure and were reminded to refrain from superfluous movements to avoid muscle contamination of the EEG data. All participants completed a 1.5 min familiarization block encompassing three repetitions per condition. This block was repeated if participants did not comprehend the goal of the BTT after the initial familiarization block. Subsequently, participants performed four blocks of the BTT, separated by short ~2 min breaks to avoid fatigue. During these four blocks, EEG data were collected (cf., Section 2.3. EEG data acquisition and preprocessing). Each block contained 13 trials per condition. The order of the trials was pseudo-randomized under the premise that all conditions should precede one another to an equal extent. In total, 156 trials were performed per participant (4 blocks × 3 conditions × 13 trials). As each block lasted 5 min and 12 s, total BTT performance lasted 20 min and 48 s.

### 2.3. EEG Data Acquisition and Preprocessing

A 64-channel EEG system (BioSemi ActiveTwo (Biosemi, Amsterdam, The Netherlands)) was used, with matching electrode cap positioned according to the 10–20 system. Data were acquired at 2048 Hz, and preprocessed offline in MATLAB (2021a, The MathWorks Inc., Portola Valley, CA, USA) (cf., Appendix A) [50]. Subsequently, EEG data were time-locked to BTT execution stage onset, and epochs were created from −3–3.5 s, with 0 s being execution stage onset. Data for motor planning were analyzed in the −2–0 s window, whereas data for motor execution were analyzed in the 1–3 s window. In line with [21], the first second of motor execution was not analyzed to avoid movement initiation artefacts.

### 2.4. EEG Time-Frequency Decomposition

MRBD during the execution and planning of (interlimb) movements has been clearly demonstrated in the past in the frontal, sensorimotor and posterior parietal brain areas in the past [16,17,18,20,51,52]. Therefore, in line with the established hypotheses and consistent with our previous work, the electrodes of interest were F3, F4, C3, C4, P3, and P4. Per electrode of interest, the cue-locked epochs were decomposed into a time-frequency representation via complex Morlet wavelets (cf., Appendix A). Power values obtained from the time-frequency decomposition were dB normalized, with baseline being the frequency-specific condition-average power values from −2.5 s to −2.2 s.

### 2.5. Statistical Analyses

RStudio (lme4 package) was used for all analyses [53,54,55]. If a linear mixed effects model was used, normality of the residuals was assessed via the Q-Q plot. For all tests, the significance level was set to α = 0.05. For all (generalized) linear mixed effects models, backward stepwise model building was performed to obtain parsimonious models. Significant effects were interpreted via pairwise contrasts between all potential pairs, corrected via the Benjamini-Hochberg False Discovery Rate procedure [56].

#### 2.5.1. Bimanual Performance in Older and Younger Adults (Hypothesis 1)

To test the hypothesis that older adults performed worse on the BTT and performance decreased with increasing complexity, a generalized linear mixed model was constructed with an Inverse Gaussian distribution and Identity link [57]. Other distributions and link functions were also explored, with the aforementioned combination resulting in the lowest AIC value. TRACKING ERROR was used as dependent variable and CONDITION (1:1, 1:3, or 3:1), GROUP (older or younger adults), and CONDITION×GROUP were included as fixed effects. BLOCK (1, 2, 3, or 4) was included as covariate fixed effect, and PARTICIPANT was included as random intercept.

#### 2.5.2. Effect of Age, Hemispheric Laterality, and Complexity on MRBD during Bimanual Planning and Execution (Hypotheses 2–4)

A grand-average time-frequency matrix was obtained by averaging the power values of the time-frequency matrixes of all participants, electrodes and BTT conditions (Appendix B, Figure A1). This grand-average matrix, which was blind to all factors of interest, was used to create a mask for data extraction purposes. To mitigate selection biases that may be introduced by means of visual mask creation, the mask was obtained by comparing each power value within the time intervals of interest (i.e., −2–0 s for planning and 1–3 s for execution) against the distribution of power values in the rest stage (−3–−2 s), using t-tests with a significance threshold of 5.008 × 10^−7^ [= 0.05/(frequencies obtained by time-frequency decomposition×matrix time-points)] [18]. Beta power values with *p*-values lower than this threshold were included in the mask (Appendix B, Figure A1). The average MRBD value within the mask was separately extracted for the motor planning and execution stage per participant, electrode and BTT condition and used for the subsequent analyses.

To test the hypotheses concerning group, hemispheric dominance and bimanual movement complexity during the planning or execution of bimanual movements, two linear mixed effects models were constructed with either MRBD during motor planning or motor execution stage as dependent variable. In both models, CONDITION (1:1, 1:3 or 3:1), GROUP (younger or older adults), HEMISPHERE (left or right) and REGION (frontal, central or parietal) served as fixed effects. All interactions, up to the 4-way interaction, were initially included in the model. Participant was included as random intercept.

#### 2.5.3. Association between MRBD and Bimanual Coordination (Hypothesis 5)

To test the hypothesis about the relationship between MRBD and bimanual coordination, a linear mixed effects model was constructed. TRACKING ERROR was included as dependent variable, and MRBD in the LEFT and RIGHT FRONTAL, CENTRAL, and PARIETAL REGIONs during both MOTOR PLANNING and EXECUTION were included as fixed effects. Additionally, GROUP and CONDITION were included as fixed effects. The interactions between MRBD-related variables and GROUP and CONDITION were included, as well. Participant was included as random intercept.

## 3. Results

All results are displayed as mean ± standard deviation. Only significant results are reported in the main text, while figures provide a more comprehensive overview.

### 3.1. Bimanual Performance in Older and Younger Adults (Hypothesis 1)

**Figure 2:**
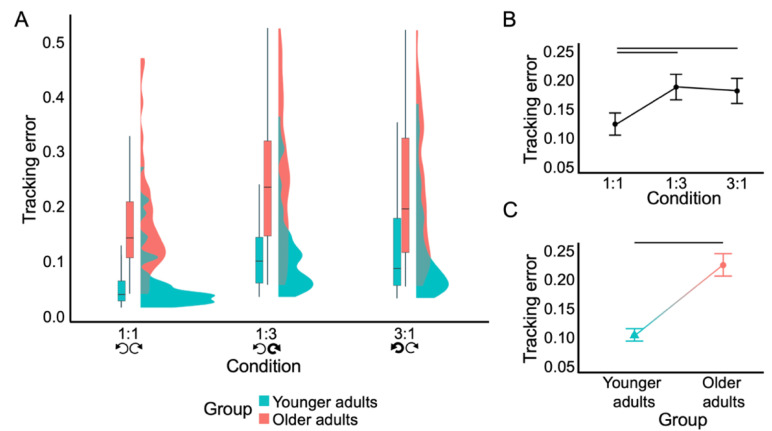
Performance on bimanual tracking task. The tracking error represents compliancy with the imposed movement condition, with a lower value representing better performance. (**A**). Box- and violin plots showing tracking error distribution per age group and condition. Whisker length is 1.5 × interquartile value. (**B**). Effect of condition on tracking error. (**C**). Effect of group on tracking error. Errors bars in (**B**,**C**) denote 95% confidence intervals, horizontal black lines denote significant post-hoc contrasts.

The final generalized linear mixed model contained CONDITION (χ^2^_2_ = 148.48, *p* < 0.001) and GROUP (χ^2^_1_ = 20.50, *p* < 0.001), but not their interaction (*p* = 0.344) (Figure 2). BTT performance decreased with increasing task complexity, as tracking error was lower in the 1:1 (0.123 ± 0.120 units) versus 1:3 (0.187 ± 0.138 units) (*z* = −9.390, *p* < 0.001) and 3:1 (0.180 ± 0.136 units) conditions (*z* = −8.495, *p* < 0.001). Moreover, tracking error was higher in older (0.224 ± 0.149 units) versus younger adults (0.103 ± 0.082 units) (*z* = 5.094, *p* < 0.001). Overall, these results corroborate Hypothesis 1 that bimanual performance is impaired in older as compared to younger adults, irrespective of condition, and decreases with increasing complexity, irrespective of age.

### 3.2. Effect of Age, Hemispheric Laterality, and Complexity on MRBD (Hypotheses 2–4)

**Figure 3:**
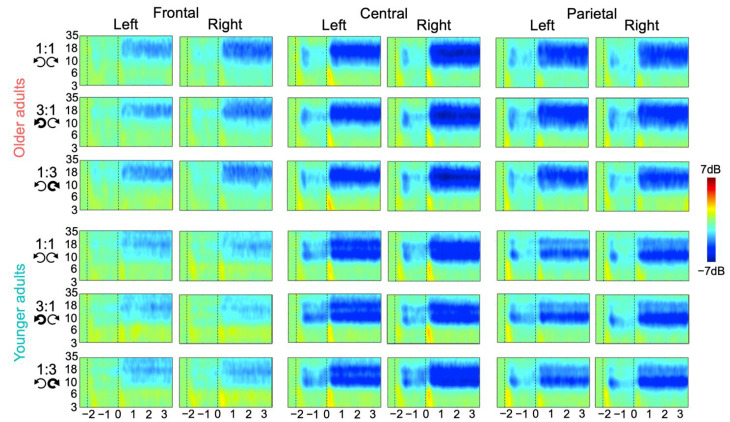
Time-frequency plots per group, condition, region, and hemisphere. The y-axis displays frequency (3–35 Hz), the x-axis displays time (−2.5–3.5 s), and the color scale displays power (−7–7 dB), with blue colors in the beta-range (13–30 Hz) reflecting movement-related beta desynchronization. Vertical dashed lines denote onset of the planning (−2 s) and execution stage (0 s).

**Figure 4:**
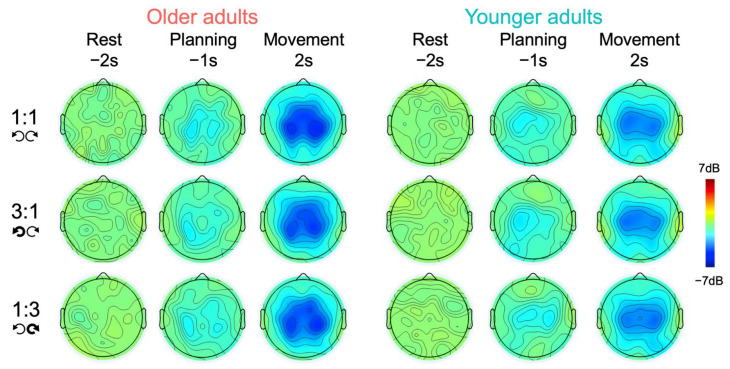
Topographic plots of spectral beta activity during rest, motor planning and execution in both older and younger adults for all three task conditions. The color scaling displays power (−7–7 dB), with blue colors reflecting movement-related beta desynchronization.

**Figure A2:**
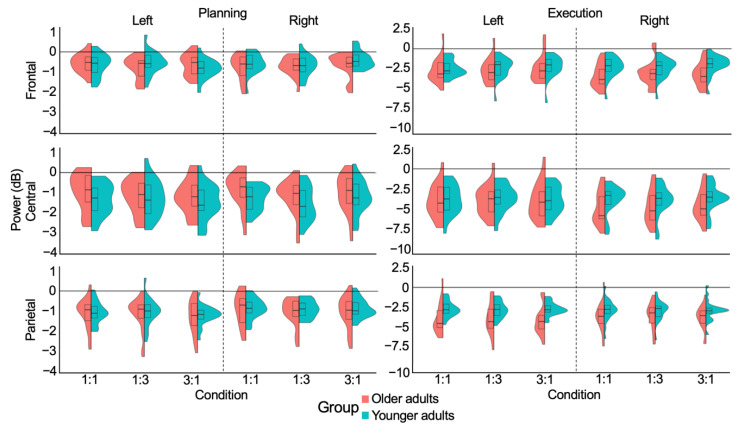
Violin plots of beta power per stage, condition, region, and hemisphere. Lower power values denote higher movement-related beta desynchronization.

The obtained time-frequency plots per condition, group, region, and hemisphere are shown in Figure 3. Figure 4 displays the topographic plots of MRBD during rest, motor planning and execution, per age group and condition. Visual inspection of these figures suggests that MRBD was mostly bound to the central region, slightly more lateralized to the left hemisphere during motor planning, and slightly more lateralized to the right hemisphere during motor execution. Additionally, an age-related MRBD increase seems observable during movement execution, but not during planning. Appendix B, Figure A2 displays all the quantitative spectral results, regardless of significance, for the sake of transparency.

#### 3.2.1. Motor Planning

**Figure 5:**
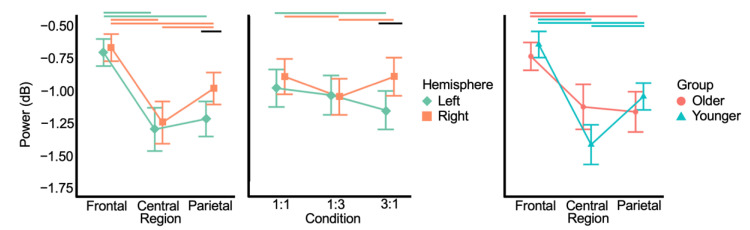
Interaction plots for movement-related beta desynchronization (MRBD) during motor planning. Lower power values represent more MRBD. In line with Hypothesis 3, MRBD was more prevalent in the left hemisphere. Notably, no hemisphere×group effect was present, contrary to Hypothesis 4. Error bars denote the 95% confidence interval, horizontal lines denote significant post-hoc contrasts with colors indicating within hemisphere/group differences and black lines indicating hemisphere / group differences.

The grand-average time-frequency mask, shown in Appendix B, Figure A1, included 60.8% of all beta power values in the planning stage. The linear mixed effects model contained HEMISPHERE (*F*_1,651_ = 8.608, *p* = 0.004), REGION (*F*_2,651_ = 93.956, *p* < 0.001), CONDITION (*F*_2,651_ = 2.984, *p* = 0.051), GROUP (*F*_1,37_ = 0.017, *p* = 0.897), HEMISPHERE×REGION (*F*_2,651_ = 3.301, *p* = 0.038), HEMISPHERE×CONDITION (*F*_2,651_ = 4.205, *p* = 0.015) and REGION×GROUP (*F*_2,651_ = 13.879, *p* < 0.001). No other effects reached the significance threshold (all *p* > 0.09). Figure 5 shows the interaction plots.

Concerning HEMISPHERE×REGION, MRBD was higher in the centroparietal regions versus the frontal regions. Namely, left frontal MRBD (−0.70 ± 0.57 dB) was lower than left central (−1.29 ± 0.91 dB) (*t* = 9.595, *p* < 0.001) and left parietal MRBD (−1.21 ± 0.74 dB) (*t* = 8.385, *p* < 0.001). Right frontal MRBD (−0.66 ± 0.57 dB) was lower than right central (−1.24 ± 0.89 dB) (*t* = 9.224, *p* < 0.001) and right parietal MRBD (−0.98 ± 0.67 dB) (*t* = 5.078, *p* < 0.001). Additionally, MRBD was generally higher in left hemisphere, with left parietal MRBD being significantly higher than right parietal MRBD (*t* = −3.782, *p* < 0.001). Lastly, right parietal MRBD was lower than right central MRBD (*t* = 4.146, *p* < 0.001).

Concerning REGION×GROUP, only within-group effects remained significant after multiple comparison correction. In older adults, frontal MRBD (−0.73 ± 0.57 dB) was lower than central (−1.12 ± 0.94 dB) (*t* = 6.189, *p* < 0.001) and parietal MRBD (−1.15 ± 0.84 dB) (*t* = 6.800, *p* < 0.001). In younger adults, frontal MRBD (−0.64 ± 0.56 dB) was lower than central (−1.41 ± 0.85 dB) (*t* = 12.706, *p* < 0.001) and parietal MRBD (−1.04 ± 0.57 dB) (*t* = 6.056, *p* < 0.001), and parietal MRBD was lower than central MRBD (*t* = 6.663, *p* < 0.001). These results indicate that MRBD during bimanual movement planning is more widespread in older versus younger adults.

Concerning HEMISPHERE×CONDITION, left MRBD was lower during the 1:1 (−1.00 ± 0.78 dB) versus 3:1 condition (−1.16 ± 0.79 dB) (*t* = 2.466, *p* = 0.043). Right MRBD was higher during the 1:3 (−1.06 ± 0.75 dB) versus 1:1 (−0.91 ± 0.73 dB) (*t* = −2.471, *p* = 0.043) and 3:1 (−0.91 ± 0.79 dB) (*t* = −2.457, *p* = 0.043) conditions. Right MRBD during the 3:1 condition was lower than left MRBD during the same condition (*t* = 3.865, *p* < 0.001).

In summary, during motor planning, we did not find an increase in MRBD in older as compared to younger adults as postulated in Hypothesis 2. The current results did align with Hypothesis 3, as MRBD was higher in the left hemisphere during motor planning. Notably, no GROUP×HEMISPHERE interaction was present, indicating no reduced hemispheric dominance in older relative to younger adults, contradicting Hypothesis 4.

#### 3.2.2. Motor Execution

**Figure 6:**
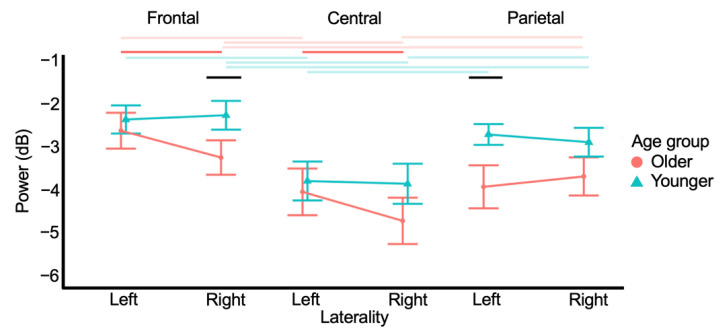
Interaction plots for movement-related beta desynchronization (MRBD) during motor execution. Lower values represent more MRBD. MRBD was higher in older adults, consistent with Hypothesis 2. Partially in line with Hypothesis 3, MRBD was higher in the frontal and central right versus left regions in older adults. Opposed to Hypothesis 4, hemispheric laterality was only present in older adults. Error bars display the 95% confidence interval. Horizontal lines denote significant contrasts with colored and black lines indicating within-group and between-group differences, respectively. Between-hemisphere significant contrasts are visualized brighter than between-region contrasts.

The grand-average time-frequency mask included 100% of all potential beta power values in the execution stage (Appendix B, Figure A1). After stepwise backward model building, the linear mixed effects model contained HEMISPHERE (*F*_1,653_ = 5.533, *p* = 0.019), REGION (*F*_2,653_ = 98.858, *p* < 0.001), GROUP (*F*_1,37_ = 5.533, *p* = 0.068), HEMISPHERE×REGION (*F*_2,653_ = 1.977, *p* = 0.139), HEMISPHERE×GROUP (*F*_1,653_ = 3.193, *p* = 0.074) REGION×GROUP (*F*_2,653_ = 2.717, *p* = 0.067), and HEMISPHERE×REGION×GROUP (*F*_2,653_ = 4.572, *p* = 0.012). All other effects, including the CONDITION effect, were not significant (all *p* > 0.68). Figure 6 shows the interaction plots.

In older adults, MRBD was highest in the central or centroparietal region, depending on the hemisphere. Namely, right central MRBD (−4.684 ± 2.028 dB) was higher than right frontal (−3.23 ± 1.51 dB) (*t* = −6.899, *p* < 0.001) and parietal MRBD (−3.67 ± 1.67 dB) (*t* = −4.800, *p* < 0.001). Additionally, left central (−4.01 ± 2.04 dB) and left parietal MRBD (−3.91 ± 1.88 dB) were higher than left frontal MRBD (−2.61 ± 1.57 dB) (*t* = 6.664, *p* < 0.001 and *t* = 6.200, *p* < 0.001, respectively). Additionally, in older adults, right frontal and central MRBD were higher than the left frontal (*t* = −2.957, *p* = 0.007) and central MRBD (*t* = −3.192, *p* = 0.004), respectively.

Within the younger adults, MRBD was highest in the central region in both hemispheres. Namely, right frontal MRBD (−2.25 ± 1.30 dB) was lower than right central (−3.82 ± 1.80 dB) (*t* = 7.674, *p* < 0.001) and parietal MRBD (−2.88 ± 1.29 dB) (*t* = 3.067, *p* = 0.005), and right central MRBD was higher than right parietal MRBD (*t* = −4.607, *p* < 0.001). Additionally, left central MRBD (−3.76 ± 1.74 dB) was higher than left frontal (−2.35 ± 1.26 dB) (*t* = −6.880, *p* < 0.001) and parietal MRBD (−2.67 ± 0.94 dB) (*t* = −5.166, *p* < 0.001). Notably, no between-hemisphere contrasts were significant in the group of younger adults.

Between groups, MRBD was higher in older compared to younger adults in the right frontal (*t* = −2.282, *p* = 0.046) and left parietal regions (*t* = −2.822, *p* = 0.013).

Summarizing, MRBD was higher in older adults during motor execution, as formulated in Hypothesis 2. Corroborating Hypothesis 3, MRBD was higher in the right versus the left hemisphere during motor execution. However, this was only the case for the frontal and central regions in older adults. Remarkably, we found increased hemispheric lateralization of MRBD in older versus younger adults, which is the opposite of what was hypothesized in Hypothesis 4.

### 3.3. Association between MRBD and Bimanual Coordination (Hypothesis 5)

**Figure 7:**
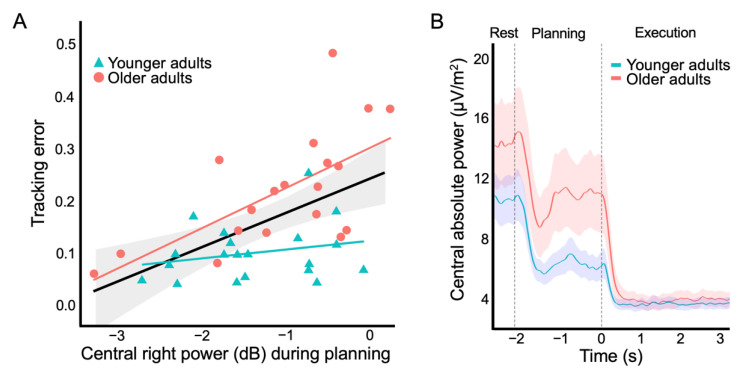
Functional role of movement-related beta desynchronization (MRBD). (**A**). Correlation between central right motor planning MRBD and tracking error. Better performance (lower error) is associated with higher MRBD. The black line shows the average correlation (ρ = 0.49, *p* = 0.002), the blue and red lines show the correlations for younger (ρ = 0.14, *p* = 0.551) and older adults (ρ = 0.56, *p* = 0.014). (**B**). Absolute central power (post-Laplacian transformation), during the bimanual tracking task (BTT). This figure aids mechanistic understanding of MRBD. Here, MRBD is the reduction of beta power at a specific timepoint during motor planning or motor execution, relative to beta power during rest.

CONDITION (*F*_2,77_ = 30.555, *p* < 0.001), GROUP (*F*_1,36_ = 19.308, *p* < 0.001) and CENTRAL RIGHT PLANNING MRBD (*F*_1,109_ = 9.806, *p* = 0.002) remained significant predictors for bimanual coordination performance after stepwise model building. All other effects were not significant (all *p* > 0.12). The effect of CONDITION and GROUP on bimanual task performance has been discussed earlier (cf., Section 3.1. Bimanual performance in older and younger adults (Hypothesis 1)). Concerning CENTRAL RIGHT PLANNING MRBD, every 1 dB MRBD decrease was associated with a tracking error increase of 0.031 units (i.e., more MR DB was associated with better bimanual task performance, irrespective of GROUP). Additionally, we calculated and visualized Spearman’s rank correlations between CENTRAL RIGHT PLANNING MRBD and TRACKING ERROR, for each age group separately, and averaged across the conditions (Figure 7A). In older adults, a moderate significant correlation between tracking error and CENTRAL RIGHT PLANNING MRBD was present (ρ = 0.56, *p* = 0.014). In younger adults, no correlation was present (ρ = 0.14, *p* = 0.551). Although the CENTRAL RIGHT PLANNING MRBD×GROUP interaction effect was not significant (*p* = 0.237), this exploratory additional correlation analysis suggests that the positive relationship between MRBD and bimanual performance was mainly driven by older adults.

Summarizing, the current results largely agree with Hypothesis 5, which stated that absolute MRBD would be positively correlated with complex bimanual coordination in older adults.

We plotted non-baseline normalized, raw, central beta power in Figure 7B. Although the subsequent interpretation is solely based on qualitative inspection, it can improve our mechanistic understanding of MRBD. In line with previous research [6,7,58], beta levels during rest are elevated in older compared to younger adults. Strikingly, during motor planning, both age groups demonstrate a similar reduction in beta power (i.e., PLANNING MRBD). To end up at the same beta power level as younger adults during motor execution, older adults then demonstrate a steep beta power decay (i.e., EXECUTION MRBD) at movement execution onset.

## 4. Discussion

Here, we examined MRBD underlying continuous bimanual movements of varying complexity in two age groups. We differentiated between motor planning and execution, and took hemispheric laterality, regionality, and movement complexity into account.

### 4.1. Beta Desynchronization during Bimanual Motor Planning and Execution

MRBD was present during both motor planning and execution in both groups, arguing in favor of a composite nature of MRBD [15,16,59]. Remarkably, MRBD differed across task conditions in the planning stage, but not the execution stage. Moreover, only during planning was a significant relationship between MRBD and bimanual coordination performance present. Previously, Tzagarakis et al. (2010) found that MRBD during motor planning was significantly affected by the amount of uncertainty about an upcoming unimanual movement, with less uncertainty being associated with higher MRBD [60]. Likewise, Doyle et al. (2005) observed that MRBD during motor planning of a discrete lateralized reaction time task depended on the amount of received information [61]. Specifically, they observed that when individuals received information about the laterality of unimanual movements, MRBD during planning in the hemisphere contralateral to the movement was enhanced, whereas MRBD during planning across both hemispheres was identical when no information about laterality was supplied. Finally, Zaepffel et al. (2013) found that only MRBD during motor planning, and not execution, was sensitive to the type of grip movement [15]. 

Through the use of a continuous bimanual task and the inclusion of two distinct age groups, our results build further on these studies that emphasize the composite, functionally polymorphic nature of MRBD. Specifically, our results suggest that MRBD encodes movement-specific processes during motor planning, but reflects more general motor processes during motor execution. Additionally, our results imply that MRBD during movement planning is particularly important for older adults, who seemed to drive the significant association between MRBD during planning and bimanual task performance (Figure 7A).

Although allocating a composite, functionally polymorphic nature to MRBD (cf., the previous paragraph and [15,60,61]) might facilitate explanation of numerous MRBD findings, it also poses several interpretational pitfalls. For instance, a functional polymorphic nature makes it cumbersome to state that MRBD during motor execution solely reflects general motor processes. It might be that movement-specific aspects are also encoded in MRBD during execution, but are overruled by the more dominant processes related to general motor execution. That being said, the observation that MRBD during motor execution is indifferent to movement types is not novel [62], and has led to the view that MRBD is a rigid mechanism that characterizes the loss of inhibition [4,8]. The current work nuances this, indicating that differentiation between MRBD during motor planning and execution is advisable, and that MRBD during planning contains movement-specific information that is capturable by EEG. Notably, this latter observation might be a promising exploit for future brain-computer interfaces to ameliorate performance.

### 4.2. Bimanual Movement-Related Beta Desynchronization Is Higher in Older Compared to Younger Adults

MRBD during bimanual motor execution was enhanced in older adults, likely to cope with elevated resting state beta power (Figure 7B and [6,7]). Remarkably, during motor planning, said age-related MRBD increase was absent (Figure 5). Instead, both groups exhibited similar MRBD values, leading to approximately the same age-related absolute difference in beta power during motor planning versus rest (Figure 7B). At first glance, the current results pertaining to MRBD and task performance may seem confusing. While both groups significantly differed in bimanual task performance and MRBD during motor execution, they did not in MRBD during motor planning. However, only MRBD during motor planning was related to bimanual task performance. We propose two mutually exclusive explanations.

First, it might be that while MRBD during planning is associated with behavioral performance, it is unrelated to the age-related deterioration of behavioral performance. While this explanation concurs with the observation that there was no GROUP×MRBD interaction in the final linear mixed model (cf., Section 3.3. Association between MRBD and bimanual coordination (Hypothesis 5)), it contradicts Blais et al. (2014), our previous work on interlimb coordination and neural oscillations, and the current exploratory analyses (Figure 7A) [5,18].

Second, the exploratory analysis gauging the relationship between task performance and MRBD during motor planning for both age groups (Figure 7A) suggests that older adults were driving the relationship between MRBD and performance. Therefore, it seems that older adults who can better compensate their resting state beta power levels via increased MRBD during motor planning (i.e., a steep decay in beta power during motor planning in Figure 7B), perform better than older adults who cannot. Likely, the latter group of older adults requires increased MRBD during movement execution to reach a certain beta threshold necessary for adequate movement performance [6,7], whereas the former group is already close to said threshold due to pronounced MRBD during motor planning (Figure 7A).

Unraveling which of these explanations holds true might be a promising avenue for future work. Given that MRBD is a relative measure, future work might also seek to disentangle the relationship between absolute beta power during rest, motor planning and motor execution, and task performance, next to the relationship between MRBD and task performance, which we tackled here. This would indicate whether (bimanual) task performance is mostly related to absolute power levels in a certain motor stage, or rather to modulatory capacity, which is embodied by MRBD.

Frontal MRBD was higher in older adults during motor planning and execution. This observation is in line with a wealth of studies reporting that older adults increasingly activate the frontal region during cognitive and motor behavior [18,63,64,65]. Traditionally, the age-related increase in neural activation has been interpreted in one of two ways: either dedifferentiation or compensation. While the former hypothesis states that age-related increased neural activation reflects a breakdown of brain network functional specificity with no positive effects on behavior, the later hypothesis states that increased activations are a successful compensational strategy of older adults [66]. As frontal activity did not explain BTT performance in the linear mixed effects model, the observed frontal MRBD increase in older adults seems to be indicative of age-related dedifferentiation. This concurs a large-scale fMRI study (*n* = 238) which reported that age-related frontal activity increases reflect reduced efficacy/specificity of neural activity [65]

### 4.3. Lateralized and Regional Specificity of Beta Desynchronization

During both bimanual motor planning and execution, MRBD was mostly situated in the centroparietal regions in older adults and the central regions in younger adults. As centroparietal electrodes better capture sensorimotor activity than frontal electrodes, this is consistent with the well-accepted view that beta dynamics are primarily present in the sensorimotor cortex [15,16,18,39].

Here, we found that MRBD was most apparent in the left hemisphere during motor planning in both age groups, and most apparent in the right hemisphere during motor execution, mainly in older adults (Figure 5 and Figure 6). Until now, research investigating lateralization of brain activity during bimanual movements has been conflicting. For instance, Rueda-Delgado et al. (2017) reported that complexity-modulated spectral beta dynamics across bimanual conditions were primarily situated in the right hemisphere [21], with Gross et al. (2005) reporting similar results [3]. On the contrary, Pollok et al. (2007) found increased beta activations in the left motor network during more complex bimanual movements [67]. Regardless, the current results are in line with our hypotheses, and are reconcilable with a wealth of research using several neuroscientific modalities to demonstrate that the left motor network is dominant during bimanual motor planning [33,34,35,36,68] and the right motor network gains dominance during bimanual motor execution [21,24,33,37,38].

Surprisingly, during motor execution, lateralization was seemingly characteristic to older adults, with only significant contrasts being present for older adults in the frontocentral regions (Figure 6). This was somewhat unexpected in light of Chettouf et al. (2020), who reported an age-related decrease in beta power lateralization during unimanual movements, and Heuninckx et al. (2005) who reported a similar age-related decrease in blood-oxygenated level dependent signal laterality during cyclical unimanual movements [31,64]. Based on the behavioral results, one might speculate that the relative task load was higher for older adults and that this gave rise to the increased laterality in older adults. However, this is contradicted by the neural results, assuming that MRBD reflects task complexity. Namely, MRBD across BTT conditions (i.e., task complexity levels) did not significantly differ during movement execution. Thus, it might be that aging differentially impacts MRBD laterality in bimanual versus unimanual movements, further emphasizing the unique character of the bimanual movements and the need for more research.

### 4.4. Beta Desynchronization and Bimanual Tracking Task

Bimanual tracking accuracy decreased with age and movement complexity. Regarding the former, an age-related decrease in bimanual task performance has been consistently reported [1,18,21,39,41]. Regarding the latter, tracking error was significantly lower in the iso-frequency (1:1) compared to the non-iso-frequency conditions (i.e., 1:3 and 3:1) in both groups. Consistent with previous work using the same task set-up and conditions, no age×complexity interaction effect was found [41]. While this might seem to contradict other studies [69,70,71], all of these studies used different BTT conditions. Likely, the 1:3 and 3:1 outward condition are not sufficiently complex to elicit an age×complexity effect, while more complex BTT conditions such as the 2:5 and 5:2 conditions do suffice [69,70,71].

We hypothesized that MRBD would increase with movement complexity (i.e., higher MRBD in the non-iso-frequency conditions). Surprisingly, no effect of bimanual task complexity was present during motor execution. During motor planning, however, a hemisphere-dependent effect was observed. Namely, MRBD in the left hemisphere was higher in the 3:1 versus 1:1 condition, and MRBD in the right hemisphere was higher in the 1:3 versus 1:1 and 3:1 condition. As MRBD was more distinct in the hemisphere ipsilateral to the hand that had to move faster, it could be that elevated MRBD signifies spatiotemporal decoupling processes, i.e., the hemisphere controlling the slow limb suppressing the mirroring of the faster movements. If so, this might imply that the previously observed MRBD increase during more complex conditions did not solely reflect increased motor activation due to increased complexity, but also reflected increased neural decoupling of otherwise coupled limbs [5,18].

**Figure A3:**
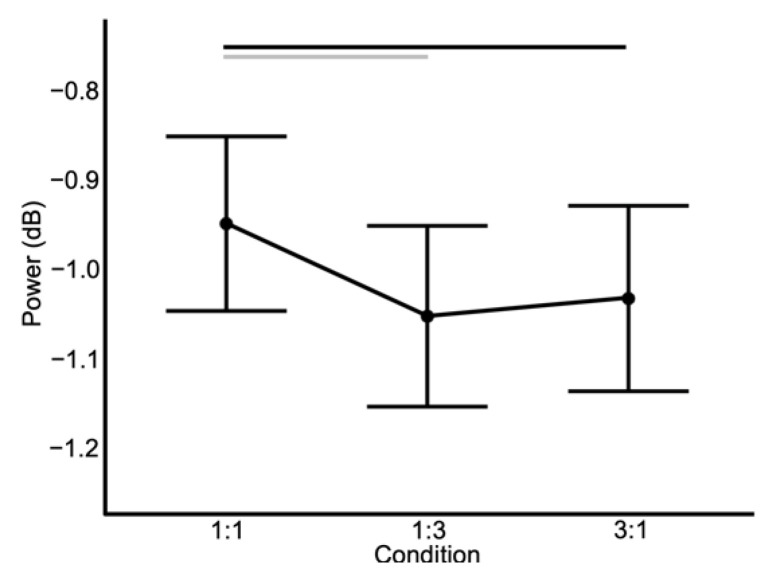
Interaction profile plot for effect of complexity on beta power during movement planning, irrespective of laterality. This plot was made for the sake of comparability with previous research. Black lines denote significant contrasts (*p* < 0.050), gray lines denote marginally significant contrasts (*p* = 0.079).

The effect of movement complexity on MRBD related to interlimb coordination in the context of aging has been examined by two studies. Namely, Blais et al. (2014) reported a distinct effect of complexity during a repetitive discrete bimanual task, with centroparietal beta MRBD being increased in the anti- and inter-phase conditions, relative to the in-phase condition, irrespective of age [5]. We previously found distinct increases in MRBD during a discrete, non-repetitive, multilimb task with varying end-effectors (hands, forefeet, or a combination of both) [18]. Thus, previous literature seemingly provides a more straight-forward interpretation of MRBD, with higher motor demands resulting in increased MRBD. However, both studies did not take laterality into account (i.e., they averaged power values across multiple electrodes over both hemispheres). To ameliorate comparison of the current results with both studies, we also conducted an exploratory post-hoc contrast for the factor CONDITION in the mixed effect model with MRBD during movement planning as a dependent variable [5,18]. By ignoring LATERALITY, we thus mimicked the design of the previous studies. Via this approach, the current results align with previous work as a significant difference between the 1:1 (−0.95 ± 0.75 dB) and 1:3 (−1.05 ± 0.78 dB) condition (*p* = 0.019), and a marginally significant difference between the 1:1 and 3:1 (−1.03 ± 0.80 dB) condition (*p* = 0.079) was present, whereas the difference between the 1:3 and 3:1 condition was not significant (*t* =−0.590 *p* = 0.555) (Appendix B, Figure A3). This exploratory analysis shines new light on the aforementioned studies, indicating that while MRBD seems to generally increase with task complexity, hemispheric laterality is a relevant factor which was formerly underrepresented.

### 4.5. Limitations and Recommendations for Future Work

Our work is prone to some limitations. First, we included two distinct age groups. Including age as a continuous variable would provide more nuanced insights into aging processes. Second, we investigated MRBD via EEG. Although this observational approach certainly yields its merits to improve basic understanding, it is limited by its inability to demonstrate causality, which could be overcome by noninvasive brain stimulation modalities capable of targeting oscillatory activity (e.g., transcranial alternating current stimulation, oscillating transcranial direct current stimulation, and repetitive TMS). Third, we did not directly assess non-baseline transformed beta dynamics during motor planning versus execution within participants, as this fell outside the current scope. While Figure 7B provides some preliminary insights, it would be enlightening if future work could further investigate whether beta power during motor planning is proportional to beta power during motor execution. Likewise, future work could also opt to investigate the increase in beta amplitude compared to rest, following movement cessation (i.e., post-movement beta-rebound). Although the functional link between post-movement beta-rebound and motor behavior is more ambiguous than the functional link between MRBD and motor behavior [6], post-movement beta-rebound is also known to change across the lifespan and is also a hallmark feature of motor behavior.

## 5. Conclusions

We analyzed the effects of aging on regional and lateralized MRBD during bimanual movements of varying complexity levels. We observed that bimanual accuracy decreases with age and movement complexity. MRBD was mainly different across both age groups during motor execution, while only right central MRBD during motor planning was associated with behavioral performance. Although the relationship between right central MRBD during motor planning and performance was present irrespective of age, exploratory analyses suggest that older adults drove this effect to significance. This, together with the knowledge that older adults have higher resting state beta levels and both groups end up at approximately the same beta levels during motor execution, implies that better performing older adults may already reach lower beta power levels during motor planning, making it easier for them to reach a certain beta threshold required for proper motor execution. MRBD was mostly present in the left, dominant, hemisphere during planning, and in the right, non-dominant, hemisphere during motor execution, corroborating previous work using other neuroscientific approaches. Our findings not only critically improve basic understanding of one of the hallmark features of (bimanual) motor behavior in the context of the aging, but they also shine new light on previous research.

## Data Availability

Data are available via the corresponding authors upon reasonably motivated request.

## Appendix A. EEG Data Prepressing and Time-Frequency Decomposition

### Appendix A.1. Data Preprocessing

Preprocessing took place in MATLAB, using custom code based on the EEGLAB plug-in (v2021.1) [50]. Data were down-sampled to 512 Hz and 1–35 Hz forward-backward band-pass filtered with a FIR filter [72]. Noisy channels were rejected using the Clean Rawdata plug-in (v2.0), followed by Common Average Referencing [73]. Removed channels were interpolated, Independent Components Analysis was performed and via the ICLabel plugin, muscle (probability threshold for removal >0.5), oculomotor (>0.5), heart (>0.7), line noise (>0.7), channel noise (>0.7) and other (>0.7) artifacts were removed [50,74]. Specifically, 22.9 ± 3.9 and 23.5 ± 5.5 individual components were removed in younger and older adults, respectively. High amplitude data components were removed through Artifact Subspace Rejection, and a surface Laplacian was applied to minimize spurious volume conduction effects [75,76].

### Appendix A.2. Time-Frequency Decomposition

The preprocessed EEG data were convoluted with complex Morlet wavelets, defined as Gaussian-windowed complex sine waves:

$$e^{i2\pi tf}\times {\;e}^{\frac{-t^{2}}{2\times \sigma^{2}}}$$

where *i* = complex operator, *t* = time, *f* = frequency ranging from 3 to 35 in 30 logarithmic steps, and

$$\sigma =\frac{10}{2\pi f}$$

From this complex signal, frequency-specific power values were extracted at each time point using the squared magnitude of the result of the convolution

$$\mathrm{Zt}:\;Power=real{\left[z_{t}\right]}^{2}+imag{\left[z_{t}\right]}^{2}$$
